# Timely initiation of breastfeeding in Zimbabwe: evidence from the demographic and health surveys 1994–2015

**DOI:** 10.1186/s13006-020-00255-2

**Published:** 2020-02-18

**Authors:** Sanni Yaya, Ghose Bishwajit, Gebretsadik Shibre, Amos Buh

**Affiliations:** 1 School of International Development and Global Studies, Faculty of Social Sciences, University of Ottawa, 120, University Private, Ottawa, ON K1N 6N5 Canada; 2 The George Institute for Global Health, The University of Oxford, Oxford, UK; 3 School of Public Health, College of Health Sciences, Addis Ababa University, Addis Ababa, Ethiopia; 4 Interdisciplinary School of Health Sciences, Faculty of Health Sciences, University of Ottawa, Ottawa, Canada

**Keywords:** Breastfeeding, Timely initiation of breastfeeding, Global Health, Zimbabwe

## Abstract

**Background:**

Timely initiation of breastfeeding or breastfeeding within 60 min of birth has been shown to be associated with significantly lower risk of infant mortality. The World Health Organization recommends starting breastfeeding within the first hour of birth, yet many women in sub-Saharan Africa do not observe this recommendation. To date, there is limited evidence of timely initiation of breastfeeding for Zimbabwe. Therefore, we undertook this study with the aim of calculating the trend in timely initiation of breastfeeding and to explore the correlates.

**Methods:**

We used five rounds of Zimbabwe Demographic and Health Survey data conducted between 1999 and 2015. Participants were 15,923 mothers currently breastfeeding or who had a childbirth within five years preceding the surveys. Outcome variable was self-reported timing of timely breastfeeding for singleton births which was categorized as early (< 60 min), late (≥ 60 min to < 2 4 h) and very late (≥ 24 h).

**Results:**

Prevalence of timely initiation of breastfeeding was 60.3% (95% Confidence Interval [CI] 57.44, 63.02) in 1999, 66.9% (95% CI 64.32, 69.4) in 2006, 65.8% (95% CI 63.7, 67.8) in 2011 and 58.3% (95% CI 56.3, 60.4) in 2015. It increased by 27 and 22% in 2006 and 2011 compared with that of the 1999 level respectively. We found no socio-economic and gender-based differentials in the prevalence of timely initiation of breastfeeding. Compared with women aged 15–19 years old, women 25–29 and 30–34 years old had higher odds of practicing timely initiation of breastfeeding. The odds of practicing timely initiation of breastfeeding among Muslim women (adjusted odds ratio [aOR] 1.2, 95% CI 1.07, 1.36) was 20% higher when compared with Christian mothers. Women who wanted to have their last child later (aOR 0.89, 95% CI 0.81, 0.97) had 11% lower odd of practicing timely initiation of breastfeeding when compared with women who wanted children then.

**Conclusions:**

The prevalence of timely initiation of breastfeeding in Zimbabwe was 58.3% in 2015, well over the 50% target recommended by WHO for all countries to attain by 2025.

## Background

Breastfeeding is a child’s first inoculation against death, disease, and poverty, but also their most enduring investment in physical, cognitive, and social capacity [1]. In 2000, 189 heads of states signed the Millennium Declaration, committing to achieve eight development goals for their countries; the target for Millennium Development Goal (MDG) 4 was to reduce the under-five mortality rate (U5MR) by two-thirds between 1990 and 2015 [2]. To reduce U5MR, improving newborn survival is critical and breastfeeding has been proven to be a vital component for infant survival especially in developing countries [3, 4].

The World Health Organization (WHO) recommends timely initiation of breastfeeding, breastfeeding a newborn within the first hour of life [5]. Timely initiation of breastfeeding is a low cost intervention that has substantial potential to reduce neonatal and early infant morbidity and mortality [6–8]. It has been reported that timely initiation of breastfeeding reduces neonatal mortality by 19.1% [9].

However, despite the benefits of timely initiation of breastfeeding, less than 40% of infants in resource limited settings are breastfed within an hour of birth [10]. Some of the documented factors associated with failure to initiate timely breastfeeding include multiparity, caesarean section, low birthweight, mother’s low level of education, mother’s occupation, place of delivery and size of baby at birth [9, 10].

Zimbabwe is one of the resource limited countries in sub-Saharan Africa with a high fertility rate of 3.68 children per woman [11] and a high infant mortality rate of 32.7 deaths per 1000 live births [12]. In Zimbabwe, the government since independence has allocated a share of the public budget to the provision of social services particularly health and education. Maternal healthcare programs and services in the country have been integrated into the public health system and efforts have been made to provide services to the poorest Zimbabweans [13].

The government has placed a high priority on fighting infant and maternal mortality and the Zimbabwe has a policy barring government health workers from charging fees to expecting mothers [14]. Despite this, there is little information about the practice and or efforts to support the practice of timely initiation of breastfeeding as an intervention to improve child survival. In order to generate information that could help in the development of feasible and sustainable strategies to improve the practice of timely initiation of breastfeeding among women, this study assessed the prevalence and correlates of timely initiation of breastfeeding in Zimbabwe.

## Methods

### Data source

Data were obtained from five rounds of the Zimbabwe Demographic and Health Survey (ZDHS), conducted between 1994 and 2015. These five ZDHS were all designed to provide up-to-date information on fertility and child mortality levels; maternal mortality; fertility preferences and contraceptive use; utilization of maternal and child health services; women’s and children’s nutrition status; knowledge, attitudes and behaviours related to HIV/AIDS and other sexually transmitted diseases; and domestic violence. All women age 15–49, who were usual members of the selected households and those who spent the night before the survey in the selected households were eligible to be interviewed. Women were also asked about their most recent births.

The surveys used a sampling frame, a list of enumerated areas that was provided by the Zimbabwe National Statistics Agency. The survey samples were stratified samples that were selected in two stages and data were collected using standard questionnaires that had been used in previous surveys in the country. Details about the sampling method has been reported elsewhere in the final report of the survey [15].

### Variables

Study variables were categorical characteristics of 15,923 mothers 15 to 49 years, from whom data was collected. The outcome of interest was self-reported timing of timely breastfeeding for singleton births which was categorized as yes (< 60 min) and no (≥ 60 min). Independent variables included sex of child (male, female); years the DHS were conducted (1999, 2006, 2011, 2015); age group of women in years (15–19, 20–24, 25–29, 30–34, 35–39, 40–49); residence (urban, rural); religion (non-Christian, Christian); education (no education, primary, secondary, higher); wealth index (poorest, poorer, middle, richer, richest); number of children ever born (1–3, > 3), and last child wanted (wanted then, wanted later, wanted no more).

### Data analysis

We analysed data using STATA version 12. To adjust for the survey design, we used the complex survey module (svyset) to account for primary sampling units, sample strata and sample weight, and all the analyses are made with these design elements accounted. The characteristics of participants were analysed using descriptive statistics, namely frequencies and percentages. Cross tabulations were performed to measure the crude prevalence of timely initiation of breastfeeding and the distribution of the outcome variable across women’s sociodemographic/economic variables for all the pooled data used in the study. The significance of association of women’s demographic/economic variables with timely initiation of breastfeeding was tested using chi-square tests.

The final step was a logistic regression analysis that assessed the adjusted associations of timely initiation of breastfeeding with women’s sociodemographic characteristics. All Variables were statistically significantly associated (*p* - value < 0.001) in the chi-square statistics and were selected for the regression analysis. The outcomes of the regression analysis were reported in terms of adjusted odd ratios and corresponding 95% CI. Variables whose 95% confidence intervals did not include the null (1.0) were considered to have a statistically significant association with timely initiation of breastfeeding. To measure change in timely initiation of breastfeeding across the five surveys, we included the year dummy in the regression analysis together with the other covariates. The estimated coefficients on included time dummies corresponding to the years 2006, 2011 and 2015 are estimates of the difference between the intercept in these periods and the intercept in the omitted period (1999). Using the 95% CI approach, we then determined whether timely initiation of breastfeeding changed with time in the course of more than two decades net of effect of other covariates. The CIs for two or more survey periods crossing the null value indicates that no change existed in timely initiation of breastfeeding between these surveys.

## Results

### Sample characteristics and breastfeeding initiation behaviors

Table 1 shows the characteristics of the surveyed women. The largest group of women (27.9%) in the study were aged 20–24 years and majority of the women (67.9%) were resident in rural areas with 11,902 (74.7%) of them being Christians. Nearly 60 % of the women (59%) had attained only the secondary level of education and 11,554 (72.6%) of the women had between 1 and 3 children, 41.3% of whom came from households with richer or higher wealth quintiles.

**Table 1:** Sample characteristics of women in Zimbabwe from 1994 to 2015 (*n* = 13,574)

Variables	Frequency (%)
Age	
15–19	1256 (7.9)
20–24	4440 (27.9)
25–29	4309 (27.1)
30–34	3060 (19.2)
35–39	1831 (11.5)
40–44	812 (5.1)
45–49	214 (1.3)
Residence	
Urban	5113 (32.1)
Rural	10,810 (67.9)
Educational level	
No Education	474 (3)
Primary	5437 (34.1)
Secondary	9443 (59)
Higher	570 (3.6)
Wealth index	
Poorest	3354 (21.1)
Poorer	3082 (19.4)
Middle	2893 (18.2)
Richer	3640 (22.9)
Richest	2953 (18.5)
Religion	
Christian	11,902 (74.7)
Muslim	2055 (13)
Other	1967 (12.3)
Parity	
1–3	11,554 (72.6)
> 3	4370 (27.4)
Wanted last child	
Wanted then	10,299 (64.7)
Wanted later	4145 (26)
Wanted no more	1477 (9.3)
Sex of child	
Male	8044 (50.5)
Female	7880 (49.5)

Table 2 shows timely breastfeeding initiation practices based on various characteristics of women. Timely initiation of breastfeeding was higher in women aged 25–29 and 30–34 years, women who were Muslims, and lower among women who intended to have their last child latter (Table 2).

**Table 2:** Cross tabulation of sociodemographic characteristics and timely initiation of breastfeeding practice of women in Zimbabwe from 1994 to 2015 (*n* = 13,574)

Variables	Timely initiation of breastfeeding % (95% CI)
Year	
1999 (2729)	60.3 (57.44, 63.02)
2006 (4025)	66.9 (64.32, 69.4)
2011 (4291)	65.8 (63.7, 67.8)
2015 (4878)	58.3 (56.3, 60.4)
Age	
15–19 (1256)	60.4 (57.2, 63.5)
20–24 (4440)	62.3 (60.5, 64)
25–29 (4309)	64.4 (62.5, 66.2)
30–34 (3060)	64.1 (61.8, 66.3)
35–39 (1831)	62.4 (59.71, 65)
40–44 (812)	60.1 (56.1, 63.9)
45–49 (214)	55.1 (47.2, 62.8)
Residence	
Urban (5113)	65.6 (63.8, 67.4)
Rural (10,810)	61.5 (60, 63)
Educational level	
No education (474)	58.1 (51.7, 64.2)
Primary (5437)	62.3 (60.5, 64.1)
Secondary (9443)	63.7 (62.3, 65)
Higher (570)	58.2 (53.8, 62.4)
Wealth index	
1 (3354)	62.1 (59.7, 64.5)
2 (3082)	59.7 (57.3, 62.1)
3 (2893)	62.6 (60.44, 64.8)
4 (3640)	65 (63.1, 67)
5 (2953)	64.4 (61.9, 66.9)
Religion	
Christian (11,902)	61.7 (60.4, 63)
Muslim (2055)	67.2 (64.7, 69.6)
Other (1967)	65.1 (62.2, 67.9)
Parity	
1–3 (11,554)	63.2 (62, 64.5)
> 3 (4370)	61.8 (59.7, 63.8)
Wanted last child	
Wanted then (10,299)	64 (62.7, 65.3)
Wanted later (4145)	60.6 (58.7, 62.4)
Wanted no more (1477)	61.1 (57.2, 64.8)
Sex of child	
Male (8044)	63.2 (61.8, 64.6)
Female (7880)	62.4 (60.9, 63.9)

### Prevalence of timely initiation of breastfeeding among women in Zimbabwe from 1999 to 2015

**Fig. 1:**
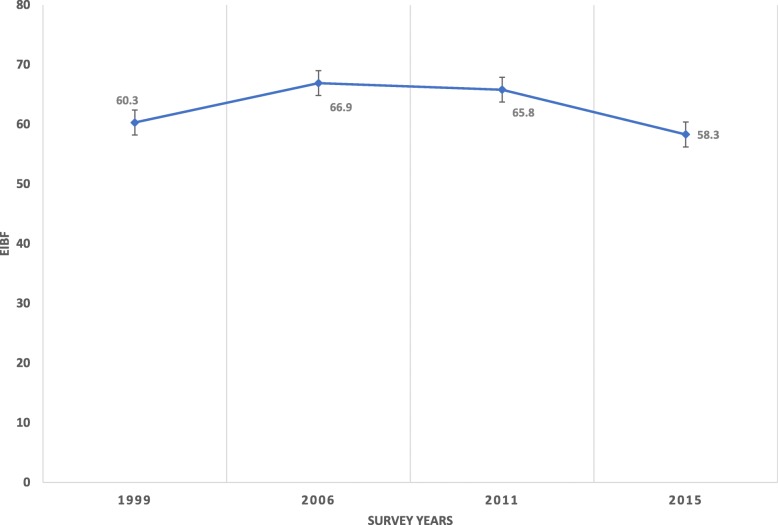
Trends in prevalence of timely initiation of breastfeeding among women in Zimbabwe by year (1994–2015)

The prevalence of timely initiation of breastfeeding among women was 60.3% (95% CI 57.44, 63.02), 66.9% (95% CI 64.32, 69.4), 65.8% (95% CI 63.7, 67.8) and 58.3% (95% CI 56.3, 60.4) respectively in 1994, 1999, 2006, 2010 and 2015. The proportion of mothers who practiced timely initiation of breastfeeding of their children have generally been on the increase between 1999 and 2011. The timely initiation of breastfeeding level in 2006 was increased by 27% compared with that of 1999, and in 2011, it increased by 22%. The prevalence of timely initiation of breastfeeding in 2015 was similar with that of the 1999 level as evidenced by overlapping CI of the estimates for the two survey years. Between 2011 and 2015, prevalence of timely initiation of breastfeeding decreased by about eight percentage points. Fig. 1 and Table 2 show the unadjusted trend without the other correlates controlled for in the model, and presented in Table 3 is the adjusted odds ratio for each of the three survey years done with the 1999 as a reference year.

**Table 3:** Correlates of timely initiation of breastfeeding among women in Zimbabwe from 1994 to 2015 (multivariable logistic regression analysis)

Variables	AOR (95% CI)
Survey year	
1999 (ref)	1
2006	1.27 (1.07, 1.51)**
2011	1.22 (1.05, 1.41)**
2015	0.89 (0.77, 1.03)
Age of the mother	
15–19 (ref)	1
20–24	1.05 (0.91, 1.22)
25–29	1.17 (1.00, 1.36)**
30–34	1.19 (1.00, 1.40)**
35–39	1.13 (0.93, 1.38)
40–44	1.07 (0.84, 1.36)
45–49	0.88 (0.62, 1.24)
Place of residence	
Urban (ref)	1
Rural	0.86 (0.73, 1.00)
Education	
No education (ref)	1
Primary	1.18 (0.91, 1.52)
Secondary	1.15 (0.89, 1.49)
Higher	0.84 (0.61, 1.16)
wealth index	
Poorest (ref)	1
2	0.899 (0.795, 1.02)
3	1.01 (0.89, 1.15)
4	1.04 (0.88, 1.23)
Richest	0.99 (0.80, 1.22
Religion	
Christian (ref)	1
Muslim	1.2 (1.07, 1.36)**
Other	1.12 (0.98, 1.27)
Parity	
1–3 (ref)	1
> 3	0.99 (0.87, 1.11)
Child wanted	
Wanted then (ref)	1
Wanted later	0.89 (0.81, 0.97)**
Wanted no more	0.86v (0.73, 1.01)
Sex of child	
Male (ref)	1
Female	0.96 (0. 89,1.03)

** = *P* - value < 0.05

### Correlates of timely initiation of breastfeeding among women in Zimbabwe (multivariable logistic regression analysis)

Results of the multivariable logistic regression analysis on the correlates of timely initiation of breastfeeding among mothers are presented in Table 3. Compared with women aged 15–19 years old, women 25–29 and 30–34 years old had higher odds of practicing timely initiation of breastfeeding. The odds of practicing timely initiation of breastfeeding among Muslim women (aOR 1.2, 95% CI 1.07, 1.36) was 20% higher when compared with Christian mothers. Women who wanted to have their last child latter (aOR 0.89, 95% CI 0.81, 0.97) had 11% lower odd of practicing timely initiation of breastfeeding when compared with women who wanted then.

## Discussion

This study assessed the prevalence, trends and correlates of timely initiation of breastfeeding) among women aged 15–49 years in Zimbabwe using the five rounds of DHS conducted between 1994 and 2015. The results reveal that the prevalence of timely initiation of breastfeeding have been on the increase between 1994 and 2011 and decreased afterwards.

The proportion of women practicing timely initiation of breastfeeding in Zimbabwe has increased between 22 and 27 percentage points between 1999 and 2011, and it fell significantly between 2011 and 2015. The finding on the timely initiation of breastfeeding in this study is comparable with that of other studies [9, 16]. However, the prevalence of timely initiation of breastfeeding in this study is lower than that of similar studies [14, 15], and higher than findings in other studies [16–19]. Also, the prevalence of timely initiation of breastfeeding reported in this study generally is well above the 50% target of timely initiation of breastfeeding recommended by WHO to be achieved by all countries by 2025 [20]. This is encouraging in the sense that the country is likely to increase the coverage of timely initiation of breastfeeding even to a higher level by the deadline.

In the regression analysis, mother’s age, religion, and their intention to have their last child latter were significantly associated with the women’s practice of timely initiation of breastfeeding. Adult women and Muslim women had higher odd of practicing timely initiation of breastfeeding, whereas women who wanted their last child later had lower odd of practicing timely initiation of breastfeeding compared with women who wanted their last child then. Many studies have reported that some factors statistically associated with the practice of timely initiation of breastfeeding include mother’s educational level, occupation, income level, age, newborn’s gender, ill-health of mother and newborn at delivery, size of newborn and perceived maternal benefits [4, 9, 20, 21].

The Zimbabwe government has allocated a share of its public budget to the provision of social services particularly health and education. Since the mid-1980s, the family planning program in the country has been integrated into the public health system and efforts have been made to provide services to the poorest Zimbabweans in an attempt to improve child and maternal survival [13]. However, little information is available concerning timely initiation of breastfeeding as a child survival intervention and its correlates in this country.

### Strengths and limitations

Unless there are studies that were done but never published, to the best of our knowledge, our study is the first to assess the prevalence and correlates of timely initiation of breastfeeding in Zimbabwe using five rounds of DHS. Our findings therefore can be useful in guiding both policy and future research on breastfeeding patterns in this country. We assessed the change in timely initiation of breastfeeding over time using one of the best approaches and hope that the finding reflects reliable evidence. Nonetheless, since the survey was self-reported, there is the possibility of recall bias. Also, given the secondary nature of the data we used, we had no control over the measurement and selection of variables which led to the omission of variables such as marital status of mother, mode of delivery, place of delivery, mother’s use of ANC services, ethnicity, mother’s ownership of a house and mother’s occupation which have been shown to influence the practice of timely initiation of breastfeeding elsewhere [9, 17, 18, 22, 23].

## Conclusions

The highest prevalence of timely initiation of breastfeeding among women in Zimbabwe since 1994–2015 is 70%, higher than the 50% target recommended by WHO for all countries to attain by 2025. The trend of timely initiation of breastfeeding saw fluctuation; it increased between 1999 and 2011, and fell afterwards. We found no differential practice of timely initiation of breastfeeding according to most characteristics of the women, except that Muslim women and women aged between 25 to 34 years had higher odd of practicing timely initiation of breastfeeding compared with their Christian and adolescent counterparts respectively. Further studies are recommended to better understand the set of factors that underlie the differential practice of timely initiation of breastfeeding and to also examine whether the same pattern of timely initiation of breastfeeding remains in different areas of the country as the DHS based findings could not be generalized to smaller areas.

## Data Availability

Data for this study were sourced from Demographic and Health surveys (DHS) and available here: http://dhsprogram.com/data/available-datasets.cfm.

## Abbreviations

AIDS: Acquired Immunodeficiency Syndrome
ANC: Antenatal care
CI: Confidence interval
DHS: Demographic and Health Survey
HIV: Human Immunodeficiency Virus
MDG: Millennium development goal
N: Frequency
OR: Odds ratio
U5MR: Under-five mortality rate
US: United States
WHO: World Health Organization
ZDHS: Zimbabwean Demographic and Health Survey

## References

[1] Hansen K (2016). Breastfeeding: a smart investment in people and in economies. Lancet.

[2] IHME. Progress towards Millennium Development Goals 4 and 5 on maternal and child mortality: an updated systematic analysis [Internet]. [cited 2018 Jul 26];Available from: http://www.healthdata.org/research-article/progress-towards-millennium-development-goals-4-and-5-maternal-and-child-mortality 10.1016/S0140-6736(11)61337-8 21937100

[3] Fombong FEE, Olang B, Antai D, Osuorah CDI, Poortvliet E, Yngve A (2016). Maternal socio-demographic determinants of exclusive breastfeeding practice in Cameroon. Am J Food Nutr.

[4] Sharma IK, Byrne A (2016). Early initiation of breastfeeding: a systematic literature review of factors and barriers in South Asia. Int Breastfeed J.

[5] WHO. Indicators for Assessing Infant and Young Child Feeding Practices Part 1 Definitions [Internet]. 2007;Available from: https://apps.who.int/iris/bitstream/handle/10665/43895/9789242596663_fre.pdf

[6] Mullany LC, Katz J, Li YM, Khatry SK, LeClerq SC, Darmstadt GL (2008). Breast-feeding patterns, time to initiation, and mortality risk among newborns in southern Nepal J. Nutr.

[7] Garcia CR, Mullany LC, Rahmathullah L, Katz J, Thulasiraj RD, Sheeladevi S (2011). Breast-feeding initiation time and neonatal mortality risk among newborns in South India. J Perinatol.

[8] Edmond KM, Kirkwood BR, Amenga-Etego S, Owusu-Agyei S, Hurt LS (2007). Effect of early infant feeding practices on infection-specific neonatal mortality: an investigation of the causal links with observational data from rural Ghana. Am J Clin Nutr.

[9] Adhikari M, Khanal V, Karkee R, Gavidia T (2014). Factors associated with early initiation of breastfeeding among Nepalese mothers: further analysis of Nepal demographic and health survey, 2011. Int Breastfeed J.

[10] Patel A, Bucher S, Pusdekar Y, Esamai F, Krebs NF, Goudar SS (2015). Rates and determinants of early initiation of breastfeeding and exclusive breast feeding at 42 days postnatal in six low and middle-income countries: a prospective cohort study. Reprod Health.

[11] Knoema. Zimbabwe - Fertility rate, 1950–2017 - knoema.com [Internet]. Knoema [cited 2018 Jul 27];Available from: https://knoema.com//atlas/Zimbabwe/topics/Demographics/Fertility/Fertility-rate

[12] Index Mundi. Zimbabwe Infant mortality rate - Demographics. [Internet]. [cited 2018 Jul 27];Available from: https://www.indexmundi.com/zimbabwe/infant_mortality_rate.html

[13] Thomas D, Maluccio J (1996). Fertility, contraceptive choice, and public policy in Zimbabwe. World Bank Econ Rev.

[14] Phiri Gift. Zimbabwe’s maternal mortality crisis. [Internet] [cited 2018 Jun 8];Available from: https://www.aljazeera.com/indepth/features/2014/02/zimbabwe-maternal-mortality-crisis-20142561739198301.html

[15] Zimbabwe National Statistics Agency and ICF International. 2016. Zimbabwe Demographic and Health Survey 2015: Final Report. Rockville, Maryland, USA: Zimbabwe National Statistics Agency (ZIMSTAT) and ICF International.. 2016 [cited 2018 May 11];Available from: http://dhsprogram.com/publications/publication-fr322-dhs-final-reports.cfm

[16] Ekubay M, Berhe A, Yisma E. Initiation of breastfeeding within one hour of birth among mothers with infants younger than or equal to 6 months of age attending public health institutions in Addis Ababa, Ethiopia. Int Breastfeed J. [Internet] 2018 [cited 2018 Aug 3];13. Available from: https://internationalbreastfeedingjournal.biomedcentral.com/articles/10.1186/s13006-018-0146-0 10.1186/s13006-018-0146-0 PMC5782370 29410699

[17] Ndirangu MN, Gatimu SM, Mwinyi HM, Kibiwott DC (2018). Trends and factors associated with early initiation of breastfeeding in Namibia: analysis of the demographic and health surveys 2000–2013. BMC Pregnancy Childbirth.

[18] Adewuyi EO, Zhao Y, Khanal V, Auta A, Bulndi LB (2017). Rural-urban differences on the rates and factors associated with early initiation of breastfeeding in Nigeria: further analysis of the Nigeria demographic and health survey, 2013. Int Breastfeed J.

[19] Issaka AI, Agho KE, Renzaho AM (2017). Prevalence of key breastfeeding indicators in 29 sub-Saharan African countries: a meta-analysis of demographic and health surveys (2010–2015). BMJ Open.

[20] Sholeye OO, Abosede OA, Salako AA (2015). Exclusive breastfeeding and its associated factors among mothers in Sagamu, Southwest Nigeria. J Health Sci.

[21] Chuwa M, Mgaya BB (2013). Factors hindering breastfeeding practices among mothers in rural Tanzania. Afr J Midwifery Womens Health.

[22] Beyene MG, Geda NR, Habtewold TD, Assen ZM (2017). Early initiation of breastfeeding among mothers of children under the age of 24 months in southern Ethiopia. Int Breastfeed J.

[23] Shiferaw BZ, Mossa KA, Gashaw BT (2017). Factors associated with early initiation and exclusive breastfeeding practices among mothers of infant’s age less than 6 months. J Pediatr Neonatal Care.

