# Correction to: Timely initiation of breastfeeding in Zimbabwe: evidence from the demographic and health surveys 1994–2015

**DOI:** 10.1186/s13006-020-00270-3

**Published:** 2020-05-04

**Authors:** Sanni Yaya, Ghose Bishwajit, Gebretsadik Shibre, Amos Buh

**Affiliations:** 1 School of International Development and Global Studies, Faculty of Social Sciences, University of Ottawa, 120, University Private, Ottawa, ON K1N 6 N5 Canada; 2 The George Institute for Global Health, The University of Oxford, Oxford, UK; 3 School of Public Health, College of Health Sciences, Addis Ababa University, Addis Ababa, Ethiopia; 4 Interdisciplinary School of Health Sciences, Faculty of Health Sciences, University of Ottawa, Ottawa, Canada

**Correction to: Int Breastfeeding J**

**http://dx.doi.org/10.1186/s13006-020-00255-2**

Following publication of the original article [1], the authors have flagged that the article contains the following errors:

1. All instances of the year ‘1994’ should instead be ‘1999’

2- The article should refer to ‘four rounds of Zimbabwe Demographic and Health Survey data’ (not ‘five rounds…’)

3- In the Conclusions of the article, it should read ‘67% higher than the 50% target’ (not ‘70% higher…’)

The authors apologize for any inconvenience caused.
